# No association of IL-12p40 pro1.1 polymorphism with juvenile idiopathic arthritis

**DOI:** 10.1186/s12969-015-0059-z

**Published:** 2015-12-15

**Authors:** Christiane S. Eberhardt, Johannes-Peter Haas, Hermann Girschick, Tobias Schwarz, Henner Morbach, Angela Rösen-Wolff, Dirk Foell, Guenther Dannecker, Carsten Schepp, Gerd Ganser, Nora Honke, Thomas Eggermann, Jan Müller-Berghaus, Norbert Wagner, Kim Ohl, Klaus Tenbrock

**Affiliations:** Département de l’enfant et de l’adolescent, Hôpitaux Universitaires de Genève, Genève, Switzerland; Deutsches Zentrum für Kinder- und Jugendrheumatologie, Garmisch-Partenkirchen, Germany; Klinik für Kinder- und Jugendmedizin, Vivantes Klinikum im Friedrichshain, Berlin, Germany; Kinderklinik der Bayerischen Julius-Maximilians-Universität, Universitätsklinikum, Würzburg, Germany; Klinik für Kinder- und Jugendmedizin, Universitätsklinikum Carl Gustav Carus, Dresden, Germany; Institut für Immunologie, Universität Münster, Münster, Germany; Pädiatrie 5, Olgahospital Stuttgart, Stuttgart, Germany; Klinik für Anästhesiologie, Klinikum der Universität Regensburg, Regensburg, Germany; Klinik für Kinder-und Jugendrheumatologie, St Josef Stift Sendenhorst, Sendenhorst, Germany; Paul-Ehrlich Institut, Langen, Germany; Institut für Humangenetik, Heidelberg, Germany; Department of Pediatrics, Division of Pediatric Pneumology, Allergology and Immunology, RWTH Aachen, University, Pauwelsstr 30, D-52074 Aachen, Germany

**Keywords:** Juvenile idiopathic arthritis, IL-12p40, IL-12B, promoter, polymorphism

## Abstract

**Background:**

IL-12p40 plays an important role in the activation of the T-cell lines like Th17 and Th1-cells. Theses cells are crucial in the pathogenesis of juvenile idiopathic arthritis. A polymorphism in its promoter region and the genotype IL12p40 pro1.1 leads to a higher production of IL-12p40. We studied whether there is a difference in the distribution of the genotype in patients with JIA and the healthy population.

**Methods:**

In 883 patients and 321 healthy controls the IL-12p40 promoter genotype was identified by ARMS-PCR.

**Results:**

There is no association of IL-12p40 pro polymorphism neither in patients with JIA compared to controls nor in subtypes of JIA compared to oligoarthritis. We found a non-significant tendency of a higher prevalence of the genotype pro1.1 in systemic arthritis (32.4 %) and in rheumatoid factor negative polyarthritis (30.5 %) and a lower pro1.1 genotype in persistent oligoarthritis (20.7 %) and in enthesitis-related arthritis (17 %). Likelihood of the occurrence of genotype IL12-p40 pro1.1 in patients with systemic arthritis (OR 1.722, CI 95 % 1.344-2.615, p 0.0129) and RF-negative polyarthritis (OR 1.576, CI 95 % 1.046-2.376, p 0.0367) compared to persistent oligoarthritis was significantly higher. This was also true for comparison of their homozygous genotypes IL-12p40 pro 1.1 and 2.2 in systemic arthritis (OR 1.779, CI 95 % 1.045-3.029, p 0.0338). However, in Bonferroni correction for multiple hypothesis this was not significant.

**Conclusion:**

A tendency of a higher prevalence of the genotype IL-12p40 pro1.1 in systemic arthritis and in rheumatoid factor negative polyarthritis was observed but not significant. Further investigations should be done to clarify the role IL-12p40 in the different subtypes of JIA.

## Background

Juvenile idiopathic arthritis (JIA) is a disease of unknown origin. Genetically susceptible individuals develop arthritis triggered by exposition to environmental factors which leads to an uncontrolled response to self-antigens. These antigens induce an initial B- or T-cellular immune reaction which results in an immune response including secretion of TNF-a, IL-6 and IL-12 and the induction and activation of Th17 and Th1 cells. JIA affects about 2-20/100000 children/year and is classified according to the ILAR criteria (International League of Associations for Rheumatology) into different subtypes such as oligoarthritis, polyarthritis, systemic arthritis and arthritis with associated psoriasis or enthesitis (see reference [1] Prakken et al. for review).

Serum levels of IL-12 are higher in patients with JIA compared to healthy controls and in particular in patients with systemic arthritis and polyarthritis. In these patients serum levels of IL-12 are also significantly higher in acute and in inactive state than in patients with oligoarthritis [2] and strikingly higher compared to healthy controls (about 5 fold elevation). Gattorno et al. have shown that it is particularly the subunit IL-12p40 and not the total IL-12 concentration that is elevated in patients with JIA. IL-12p40 expression by activated B cells has also been documented in the inflamed joints of JIA patients [3]. While patients with polyarthritis or oligoarthritis in remission do not differ from healthy controls [4], serum levels of IL-12 are still found elevated in patients with systemic arthritis in remission.

IL-12p40 is encoded on Chromosome 5q31–33 [5], produced mainly by macrophages, neutrophils, microglia and dendritic cells [6] and promotes in cooperation with IL-12p35 (as IL-12p70) the induction and activation of Th1-cells while it promotes in cooperation with IL-23p19 (as IL-23) the induction and activation of Th17-cells, both key elements in the pathophysiology of JIA. An insertion/deletion polymorphism in the promoter region of IL-12p40 ((GC/(GC) _del_(CTC TAA)_ins_); rs17860508 [5]) with its two alleles IL-12p40 pro1 and IL-12p40 pro2 has an influence on the expression of IL-12p40: The genotype IL-12p40 pro1.1 leads to a higher production of IL-12p40 from PBMC in silicosis patients [7], in asthma patients [8] and in dendritic cells of healthy donors, the latter however showing no elevated secretion of IL-12p70 [9]. Given the importance of IL-12p40 in the induction of Th1 and Th17 cells and the possible importance in the pathogenesis of JIA, we hypothesized that the repartition of the IL-12p40 pro-genotype is different in children affected by JIA from healthy controls and in-between subtypes. Since this 6 bp insertion/deletion polymorphismus is not regularly tested on whole genome SNP arrays, it has not yet been identified as being important.

## Methods

### Patients

Blood DNA samples of 883 patients with juvenile idiopathic arthritis (180 with polyarthritis, 469 with oligoarthritis, 139 with systemic arthritis, 29 with psoriasis related, 52 with enthesitis related arthritis and 14 with unspecific JIA) have been collected in 3 German paediatric rheumatology departments (673 patients from the German Center of paediatric rheumatology, Garmisch–Partenkirchen, Department of Pediatrics, University Hospital Tübingen, Olgahospital Stuttgart and St Josef Stift Sendenhorst, 134 patients from the University Hospital Würzburg, 76 patients from the University Hospital Dresden). Blood DNA samples of 321 healthy subjects, unmatched for age and sex but all Caucasians from Germany, were from department of Humangenetik RWTH Aachen. Ethical approval of local institutions and informed consent according to the Declaration of Helsinki was obtained.

### PCR

Genotyping was performed by amplification refractory mutation system PCR (ARMS-PCR). The IL-12p40 promoter region was screened for two different already described alleles (GC/(GC) _del_(CTC TAA)_ins_) (rs17860508 [5]). The longer allele IL-12p40 pro1 is detected by the specific primer IL-12p40 5–8 (TGT CTC CGA GAG AGG CTC TAA), the 4 bp shorter allele IL-12p40 pro2 by IL-12p40 5–10 (TGT CTC CGA GAG AGG GCT GT). As generic 3′ primer IL12p40 3–5 (TGG AGG AAG TGG TTC TCG TAC) was used for both reactions (Primers from Eurogentec, Cologne, Germany). As control primers derived from the C reactive protein-gene CRP 3 and 5 were employed (CRP 3: CCA GCC TCT CTC ATG CTT TGG TTG GCC AGA CAG, CRP5: GGG TCG AGG ACA GTT CCG TGT AGA AGT GGA).

The reaction mix contained 10–20 ng genomic DNA, 1x PCR Buffer (Qiagen, Hilden, Germany, containing 15 mM MgCl_2_), 1.5 μl MgCl_2_ (25 mM), 0.5 μl dNTP 10 mM, 1U Top Taq DNA Polymerase (Qiagen Hilden, Germany), 5 pmol of each IL-12p40 primers and 2.5 pmol of each CRP primer. Taq activation was performed by an initial step of 95 °C (15 min), followed by 35 cycles (94 °C for 30s, 65 °C for 30s, 72 °C for 30s). PCR product was visualized in a 2.5 % agarose gel with 0.01 % ethidium bromide.

### Statistics

Chi-square test (3x2) and two-sided Fisher’s *t*-test were performed using Prism 5.0 GraphPad software. *P*-values were considered significant when < 0.05, Odds ratios are cited with their 95 % confidence interval (CI). Bonferroni correction of the *p*-value was performed for multiple hypothesis testing. *P*-values were considered significant when < 0.008.

## Results

### No significant association of IL-12p40 pro polymorphism in patients with JIA compared to healthy subjects

A genotyping of the IL-12p40 pro polymorphism was performed in 883 patients with JIA and in 321 healthy controls.

There was neither a significant difference in the occurrence of the IL-12p40 promoter genotype nor in the distribution of alleles (see Table 1). Noteworthy is a clear but non-significant tendency of a higher prevalence of the genotype 1.1 in systemic arthritis (32.4 %) and in rheumatoid factor negative polyarthritis (30.5 %). In addition we found a clearly lower pro1.1 genotype (21.75 %) in favour of a high IL-12p40 pro2.2 genotype (25.8 %) in oligoarthritis, the same was true in enthesitis-related arthritis but here in favour of a higher IL-12p40 pro1.2 genotype (61 %). However there was no significant different distribution of genotypes between all JIA patients or the subtypes of JIA compared to healthy controls (Table 1).

**Table 1:** Distribution of genotypes IL-12p40 pro 1.1, IL-12p40 pro 1.2, IL-12p40 pro 2.2 and of alleles IL-12p40 pro 1 and IL-12p40 pro 2 in patients with a subtype of JIA and healthy controls (absolute number and percentage), all in Hardy-Weinberg equilibrium

	No of patients (% of JIA)	IL-12 p40 pro1.1	IL-12 p40 pro1.2	IL-12 p40 pro2.2	Chi^2^-test *p*-value
Polyarthritis RF positive	29 (3.3 %)	6 (20 %)	12 (41 %)	11 (37.9 %)	0.2306
Polyarthritis RF negative	151 (17.1 %)	46 (30.46 %)	70 (46.36 %)	35 (23.17 %)	0.7842
Systemic arthritis	139 (15.7 %)	45 (32.4 %)	64 (46.0 %)	30 (21.6 %)	0.556
Oligoarthritis	469 (58.1 %)	102 (21,75 %)	246 (52.45 %)	121 (25,8 %)	0.1866
Psoriasis-related arthritis	29 (3.3 %)	7	15	7	0.92
Enthesitis-related arthritis	52 (5.9 %)	9 (17 %)	32 (61 %)	11 (21 %)	0.1932
Undifferentiated arthritis	14 (1.6 %)	4	7	3	
All patients with JIA	883	219 (24.8 %)	446 (50.5 %)	218 (24.7 %)	0.654
Healthy individuals	321	88 (27.4 %)	157 (48.9 %)	76 (23.7 %)	
Allele pro 1	JIA	884 (50.1 %)	0.432		
	Healthy Controls	333 (51.9 %)			
Allele pro 2	JIA	882 (49.9 %)			
	Healthy Controls	309 (48.1 %)			

Chi-square test was performed between subtypes and healthy controls

### No significant association of the predominant genotype IL-12p40 pro1.1 in patients with systemic arthritis and polyarthritis compared to oligoarthritis

Comparing the genotypes IL-12p40 pro1.1 and IL-12p40 pro2.2 of patients with oligoarthritis to other all other subtypes of JIA there was no significant difference in distribution. The significantly higher IL-12p40 pro1.1 genotype seen in systemic arthritis or polyarthritis versus oligoarthritis in single comparison did not remain significant when tested for multiple comparison with the Bonferroni method (p 0.011 for systemic arthritis, p 0.03 for polyarthritis RF negative; p significant < 0.008).

**Fig. 1:**
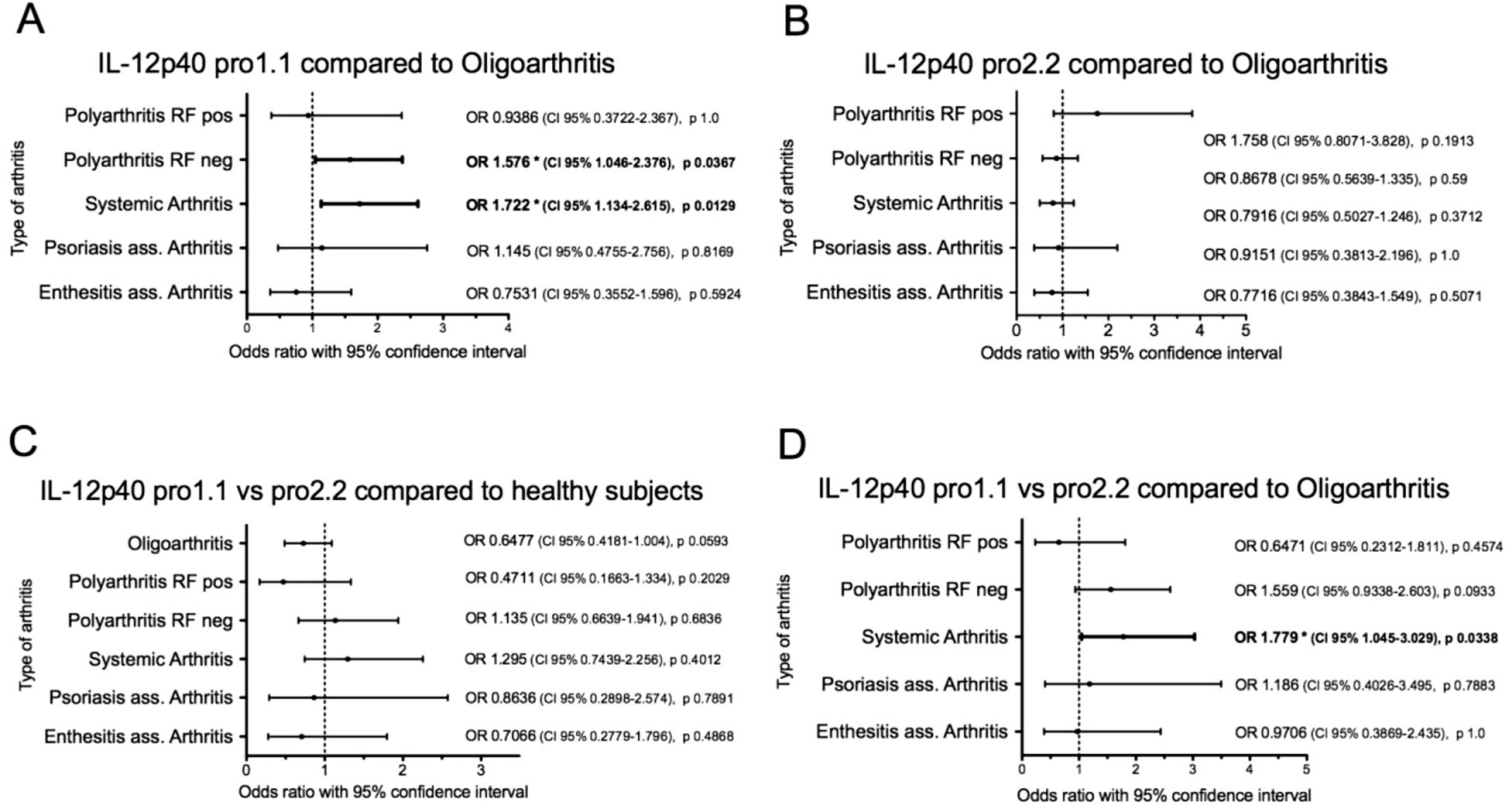
Forest plots of OR and its 95 % CI: Relation of homozygous genotypes pro 1.1 (**a**) and pro2.2 (**b**) between subtypes of JIA and oligoarthritis. Comparison of distribution of genotype IL-12p40 1.1 vs 2.2 between JIA and healthy subjects (**c**) and between subtypes of JIA and Oligoarthritis. The depicted significant Odds-ratios were not significant in Bonferroni analysis, that requires *p* < 0.008

By pooling the genotypes IL-12p40 pro1.2 and 2.2, we tested an association of IL-12p40 pro1.1 in systemic arthritis or polyarthritis versus oligoarthritis. The likelihood of the occurrence of genotype IL-12p40 pro1.1 in patients with systemic arthritis and RF-negative polyarthritis is nearly twice as high as in oligoarthritis (Fisher’s *t*-test for systemic arthritis: OR 1.722, CI 95 % 1.134-2.615, p 0.011, for polyarthritis RF negative OR 1.576, CI 95 % 1.046-2.376, p0.03, Fig. 1a). Testing of the homozygous genotypes IL-12p40 pro1.1 and pro2.2 substantiated this finding: the likelihood of prevalence of IL-12p40 pro 1.1 was 1.8 times higher in systemic arthritis (OR 1.779, CI 95 % 1.045 -3.029, p 0.034) but not for RF negative polyarthritis (OR 1.559 CI 95 % 0.934-2.603, p 0.089) compared to the distribution in oligoarthritis (Fig. 1c + d). However, again in Bonferroni correction for multiple comparison this did not remain significant (*p* < 0.008).

Furthermore, there was no association for the genotypes of children with other subtypes of JIA and oligoarthritis, neither for the genotype IL-12p40 pro2.2 (Fig. 1b) nor for rheumatoid factor negative and positive polyarthritis (Chi^2^-Test p 0.22).

## Discussion

The present study is the first to investigate the functional relevant IL-12p40 promoter polymorphism in children with JIA. There was a tendency but no clear evidence for an association between the genotypes of patients with JIA or its subgroups and healthy controls.

Regarding the genotypes of the control subjects, in this group genotype IL-12p40 pro1.1 was more frequent than genotype IL-12p40 pro2.2. In several European studies either type IL-12p40 pro1.1 was less frequent than pro2.2 (20 % versus 22-31 % [10, 11]) or nearly equal (24 % versus 26 % [8]), in other surveys type pro1.1 was more common than IL-12p40 pro2.2 (30-35 % for type pro1.1 and 15-20 % for type pro2.2, mainly Bulgarian patients [7, 12]).

These somewhat mixed results are likely influenced by the high impact of the geographical origin and the number of tested individuals on the genotype’s distribution. In this study the latter factor is statistically limiting. Therefore no tribute was paid to the lower prevalence of the IL-12p40 pro1.1 genotype in patients with oligoarthritis compared to the here tested healthy subjects since most of the European study controls have similar percentages of the pro 1.1-genotype.

A tendency of a more frequent genotype IL-12p40 pro1.1 in systemic and polyarticular arthritis compared to oligoarthritis could not be proven significant in multiple comparison testing. This is partially due to the low number of samples in the subgroups of systemic and polyarticular arthritis. Control studies in other populations are needed to definitively exclude any association of the here tested genotypes of the promoter of IL-12p40 with JIA.

Given the heterogeneity of the subgroups of JIA and the supposition of their different aetiologies [13], it is still possible that IL-12p40 is an important element in the pathogenesis of systemic and polyarticular arthritis. This is supported by studies showing a high level of IL-12 respectively IL-12p40 in the serum of patients with these two diseases in contrast to other subtypes [2, 4]. Analogically this does apply for patients with rheumatoid arthritis where no association of the IL-12p40 promoter genotype had been found [14]: In their synovial fluid the concentration of IL-12p40 was not elevated in contrast to JIA-polyarthritis and juvenile enthesitis-related arthritis [15]. Furthermore in autoimmune arthritis the presence of IL-12 is necessary to convert Th17 cells in vitro into Th17/1 cells [16]. This shift correlates with higher parameters of inflammation in patients with oligoarticular-onset JIA [17]. Therefore our primary assumption was that patients with oligoarthritis might have a higher prevalence of the IL-12p40 pro1.1 genotype. However this was not the case and might be related to the fact that the IL-12p40 pro1.1 phenotype does not necessarily result in an enhanced IL-12p70 production, which is needed for the conversion of Th17 into Th1 cells.

Therefore further studies are necessary to elucidate the exact role of IL-12p40 in JIA and its different subtypes.

## Conclusion

No clear association between the polymorphisms of the IL-12p40 promotor of JIA patients or their subtypes could be found. Control studies in other populations are needed to definitively exclude any association and further studies should be done to evaluate the exact function of IL-12p40 in JIA.

## Abbreviations

Th-cells: T-helper-cells
OR: Odds ratio
JIA: Juvenile idiopathic arthritis
